# Older adults with lower working memory capacity benefit from transcranial direct current stimulation when combined with working memory training: A preliminary study

**DOI:** 10.3389/fnagi.2022.1009262

**Published:** 2022-10-10

**Authors:** Sara Assecondi, Rong Hu, Jacob Kroeker, Gail Eskes, Kim Shapiro

**Affiliations:** ^1^Center for Mind/Brain Sciences—CIMeC, University of Trento, Rovereto, Italy; ^2^Visual Experience Laboratory, School of Psychology, University of Birmingham, Birmingham, United Kingdom; ^3^Center for Human Brain Health (CHBH), University of Birmingham, Birmingham, United Kingdom; ^4^Department of Neurology, School of Medicine, Guangzhou First People’s Hospital, South China University of Technology, Guangzhou, China; ^5^Departments of Psychiatry and Psychology and Neuroscience, Dalhousie University, Halifax, NS, Canada

**Keywords:** non-invasive brain stimulation (NIBS), tDCS—transcranial direct current stimulation, working memory training (WMT), plasticity, older adults, individual differences

## Abstract

Aging is a very diverse process: successful agers retain most cognitive functioning, while others experience mild to severe cognitive decline. This decline may eventually negatively impact one’s everyday activities. Therefore, scientists must develop approaches to counteract or, at least, slow down the negative change in cognitive performance of aging individuals. Combining cognitive training and transcranial direct current stimulation (tDCS) is a promising approach that capitalizes on the plasticity of brain networks. However, the efficacy of combined methods depends on individual characteristics, such as the cognitive and emotional state of the individual entering the training program. In this report, we explored the effectiveness of working memory training, combined with tDCS to the right dorsolateral prefrontal cortex (DLPFC), to manipulate working memory performance in older individuals. We hypothesized that individuals with lower working memory capacity would benefit the most from the combined regimen. Thirty older adults took part in a 5-day combined regimen. Before and after the training, we evaluated participants’ working memory performance with five working memory tasks. We found that individual characteristics influenced the outcome of combined cognitive training and tDCS regimens, with the intervention selectively benefiting old-old adults with lower working memory capacity. Future work should consider developing individualized treatments by considering individual differences in cognitive profiles.

## Introduction

Aging is an unavoidable and complex process that affects every person, but with an unpredictable outcome, as it is sensitive to individual differences. Some people age well, retaining most cognitive functioning, while others experience mild to severe cognitive decline that can eventually lead to a pathological condition (Cohen et al., 2019). Delaying cognitive decline with lifestyle choices, such as taking part in cognitively stimulating activities is possible but new approaches (e.g., tDCS combined with cognitive training) are needed to counteract cognitive decline once it appears (World Health Organization, 2019).

This age-related decay of cognitive function can greatly impact the independent living of individuals (Grady, 2012). Thus, it is not surprising that scientists, albeit with contradictory results (for reviews, Shipstead et al., 2012; Soveri et al., 2017a), have been searching for ways to slow or even counteract physiological cognitive decline, often focusing on working memory. Working memory is a cognitive system of limited capacity that declines with age but is crucial to many decision-making processes and everyday life functioning (Engle and Kane, 2003). Working memory training usually involves completing a certain number of sessions of an adaptive working memory task (e.g., the n-back (Kirchner, 1958) is a common choice), in which task difficulty adapts to the individual’s performance to keep it challenging, entertaining, and more effective (Harvey et al., 2018; Flegal et al., 2019; Green et al., 2019).

Individuals can learn new skills up to very old age, promoting plastic brain changes (Pauwels et al., 2018a,b). For example, training executive function can lead to structural changes (increase in gray matter and cortical volume) with a general increase in the volume of frontal and parietal brain areas (for a review, see Nguyen et al., 2019). While being directly influenced by genetic factors (Pearson-Fuhrhop et al., 2009), plasticity is also modulated by individual differences (Jones et al., 2006), such as age, education, or motivation (Barrett et al., 2004; Morais et al., 2018). Higher education has been associated with better working memory abilities (Morais et al., 2018), as has motivation (Mohammed et al., 2017). In addition, initial working memory scores can predict training effectiveness, with low working memory capacity predicting lower training gains after a dual n-back training regimen (Matysiak et al., 2019). Pathological conditions like depression and cognitive decline (Hill et al., 2000) may also impact plasticity.

Transcranial direct current stimulation (tDCS) has also been shown to modulate brain plasticity. TDCS modulates cortical excitability (Nitsche et al., 2008; Stagg and Nitsche, 2011; Kronberg et al., 2020), which in turn may boost specific plasticity, namely that of synaptic pathways activated by task performance (Bikson and Rahman, 2013) or networks with increased oscillatory activity (Reato et al., 2010, 2015). Specifically, tDCS can be highly effective when coupled with a relevant behavioral task (Kim and Ko, 2013; Lapenta et al., 2013; Pirulli et al., 2013; Filmer et al., 2014). For example, one session of 13 min of active (anodal, 1.5 mA) tDCS to the left dorsolateral prefrontal cortex (DLPFC) increased frontal activity, which correlated to enhanced working memory performance during an n-back task in older adults (Cespón et al., 2017, 2019). There was not, however, a net working memory improvement in anodal tDCS compared to the sham condition.

In the context of cognitive training with multiple sessions, researchers find that training regimens combined with tDCS can boost training and transfer gains (Au et al., 2016; Ruf et al., 2017; Cespón et al., 2018) by achieving longer-lasting maintenance of improvements (Au et al., 2017), or by shortening the training duration, without sacrificing training gains (Antonenko et al., 2018; Nissim et al., 2019). For example, older adults receiving 10 sessions of active (anodal, 1.5 mA) tDCS to the DLPFC during cognitive training maintained performance improvements for up to a month, both in the training and a transfer task (Jones et al., 2015b). Similarly, only older adults receiving bilateral tDCS of the DLPFC for 10 sessions of cognitive training maintained their performance benefits up to a month later in comparison to the sham group (Park et al., 2014).

Importantly, and leading to the rationale for the present report, the outcomes of interventions using simultaneous working memory training and brain stimulation are not always successful (Berryhill and Jones, 2012; Perceval et al., 2016; Horne et al., 2021). TDCS applications require the choice of many parameters, including the position and size of the electrodes and the duration and intensity of the stimulation current. There is also variability in the working memory training task chosen, its duration, and in the scheduling of the training (Berryhill, 2017; Hurley and Machado, 2018; Weller et al., 2020). Moreover, anatomical parameters (head anatomy, inter-, and intra-cranial volumes) that change with age determine the tDCS current distribution in the brain (Opitz et al., 2015; Antonenko et al., 2020). Finally, the initial level of performance also plays a role in the effectiveness of brain stimulation. TDCS appears to selectively benefit individuals with low baseline performance (Tseng et al., 2012; Hsu et al., 2014; Benwell et al., 2015; Wang et al., 2019), with initial capacity modulating the effectiveness of tDCS just as it modulates that of cognitive training (Matysiak et al., 2019; Krebs et al., 2021).

It has been suggested that tDCS effects are state-dependent, i.e., they are influenced by brain state at the time of stimulation (Krause et al., 2013; Krause and Cohen Kadosh, 2014; Benwell et al., 2015). We previously found (Assecondi et al., 2021) that the outcome of combined cognitive training and tDCS is sensitive to individual differences in young adults. We considered two aspects of such differences: initial performance in working memory capacity and strategy use. In particular, baseline abilities and motivation (Jones et al., 2015a; Katz et al., 2017) can contribute to shaping the outcome of combined tDCS and training protocols. To what extent these factors influence performance in older individuals remains unclear (but see Krebs et al., 2021 for a recent report) and forms the basis of the experimental question addressed in the present report.

Thus, this project aimed to assess the effectiveness of a working memory training regimen combined with tDCS in older adults. Thirty individuals were randomly assigned to one of two groups receiving either SHAM or ACTIVE stimulation, and all participants entered a 5-day training regimen using an adaptive n-back paradigm. In line with previous findings (Assecondi et al., 2021; Krebs et al., 2021), we hypothesized that individuals would respond differentially to the intervention depending on their initial individual cognitive characteristics. Specifically, we predicted that individuals with lower working memory capacity would benefit more from the combined training regimen than those with high working memory capacity.

## Materials and methods

### Participants

Two of the thirty older adults recruited withdrew because of health issues. All the remaining 28 participants (mean age 67.9 ± 6.1 yr, range 56–76, 14 females) had normal or corrected-to-normal vision, a Montreal Cognitive Assessment (MOCA) score above 26 (Nasreddine et al., 2005), no previous brain injury, no history of epilepsy or depression, were not taking medications likely to alter cortical excitability, had not received brain stimulation or cognitive training in the previous 6 months, and fulfilled safety inclusion criteria for brain stimulation (Antal et al., 2017). The study was approved by the University of Birmingham’s Ethics Committee, and informed consent was obtained before taking part in the experiment. Individuals were compensated £100. Participants were randomly allocated to one of the two stimulation groups, receiving either ACTIVE or placebo (SHAM) stimulation. Because of the age spread of recruited individuals, a posteriori participants were further split into younger older adults (YO or young-old) and older older adults (OO or old-old), based on a median split of their age (median age was 69.5 yr). This resulted in two groups (ACTIVE; SHAM) for each AGE (YO; OO).

### Transcranial direct current stimulation

2 mA tDCS was administered using an 8-channel device (Starstim, Neuroelectrics^®^) for 20 min. Two circular electrodes (NG Pistim, Neuroelectrics, area = 3.14 cm^2^), filled with conductive gel (SignaGel, ParkerLabs), were placed over the dorsolateral prefrontal cortex (DLPFC, F4 on the 10–20 system, anodal electrode), and the contralateral supraorbital site (Fp1, cathodal electrode). Impedance always remained below 20 kOhm. Both protocols (ACTIVE and SHAM) included 20 min of either 2 mA or 0 mA preceded and followed by 30 s ramping up and down (total stimulation time = 21 min). Simulations showing the distribution of the electric field and current density obtained with this procedure are reported in Supplementary material. Participants were randomly assigned to either the ACTIVE or the SHAM tDCS condition with blinding efficacy and side effects recorded *via* questionnaire.

### Working memory training: Adaptive spatial n-Back

Working memory was trained with N-IGMA (Ploughman et al., 2019), a proprietary naturalistic adaptive spatial n-Back (ANBACK) task designed to stimulate participants’ engagement (see Supplementary material for an example of the stimuli used). Participants were requested to track a series of locations and indicate whether the current location was a match or not of the location presented at “*n*” trials ago, following the method of Verhaeghen and Basak (2005). The inter-trial duration was 3 s, and participants responded by pressing the left (“match”) or the right (“no match”) arrow on the computer keyboard with their dominant hand. Each training session consisted of 20 blocks, and each participant started at “*n*” = 1 and moved to “*n*”+1 every time their performance was at or above 90% accuracy. Motivation was encouraged by feedback after every response and a short text after every block. The average “*n”* during a session ($\bar{n})$ was the dependent variable. The total duration of the training session was approximately 20 min, with variability from individual to individual due to the duration of self-paced breaks. The working memory training always started at the same time as the tDCS, with an average duration (across sessions and participants) of 21 min and 39 s (as such, the training could last a few seconds more than the stimulation).

### Outcome measures

An individual’s working memory capacity score was calculated by averaging performance in five tasks, all relying on different aspects of working memory. We used a Change Detection task (CD), a spatial (SNBACK) and a visual n-back (VNBACK), and the digit span backward (DSB) and forward (DSF). Details on how these tasks were implemented and an example of the stimuli are provided in Supplementary material.

**FIGURE 1:**
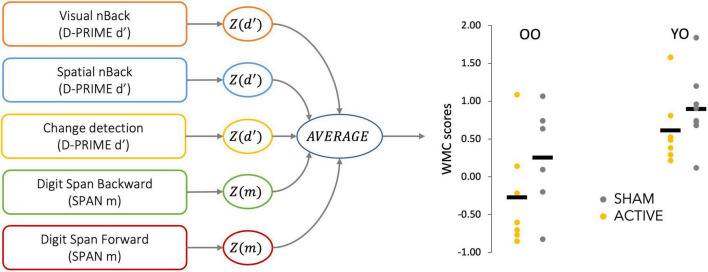
Diagram of the procedure to calculate composite working memory capacity (WMC) scores. Dependent variables measuring working memory performance were extracted from each task, standardized, and averaged, obtaining a composite score for each participant. The obtained distribution of composite scores is shown for each age and stimulation group.

First, we derived a task-dependent performance measure for each task (d-prime for the CD, the visual VNBACK, the SNBACK, and the maximum span for the DSF and DSB) at baseline and post-test. We then standardized performance separately for each task, based on the entire sample at baseline and averaged them for each person, obtaining a *composite working memory capacity score (WMC score)*^1^; see Figure 1 for a description of the procedure. Finally, we calculated changes in composite WMC scores (WMC_*POST*_-WMC_PRE_) after training.

### Questionnaires

To control for individual differences in participants’ lifestyles and attitudes that could influence the training outcome, we administered a series of questionnaires listed in Table 1. Before each training session, we asked participants about their attitude and expectation toward the intervention. After each training session, participants reported on side effects of the stimulation, and, at the end of the last session, we asked them about the strategy used during training, if any, and to guess if they were in the ACTIVE or SHAM group (to confirm the efficacy of the blinding procedure).

**TABLE 1:** Description of the questionnaires administered to participants, and where they were filled in (at home or in the lab).

Questionnaire	Description	Where
Health history (custom made)	A series of questions about past health history and current medications	Home
Hospital anxiety and depression scale (HADS; Zigmond and Snaith, 1983)	A measure of depression and anxiety	Home
Quality of life assessment (World Health Organization [WHO], 1996)	Individual’s perception of their quality of life	Home
Epworth sleepiness scale (Johns, 1991)	Habitual sleepiness	Home
Familiarity with technology	A series of questions to record subject’s familiarity with everyday technology	Home
Motivation and expectation	Feelings and attitude toward the intervention	Lab
Side effects of brain stimulation	Perceived side effects of tDCS	Lab
Strategy feedback	Use of strategy during tasks	Lab
Blinding	Perceived experimental group	Lab

### Procedure

**FIGURE 2:**
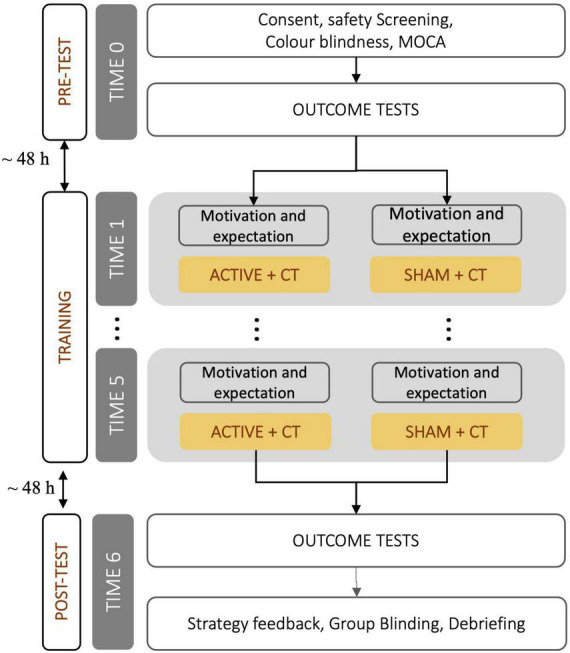
Flowchart of the procedure. CT, cognitive training.

A flowchart of the procedure is shown in Figure 2. On the first day (TIME 0—PRE) participants were invited to the lab and asked to sign a consent form. After being screened for tDCS safety (Antal et al., 2017), color blindness (Farnsworth, 1943) and cognitive impairment (MOCA; Nasreddine et al., 2005), individuals were randomly assigned to stimulation groups. Then, participants received various questionnaires to complete at home (see Table 1) and were administered the computerized outcome tests to assess baseline cognitive performance. Finally, participants familiarized themselves with the stimulation procedure and asked questions. This first session took approximately 2 h and was administered on a Friday.

After the weekend following the initial session,^2^ participants returned to the lab to start the cognitive training phase. After having the tDCS cap fitted, participants answered four questions about their mental status (level of alertness, sadness, motivation to train, and expectation on the intervention) on a continuous scale from 1 to 100, and then began 20 min of training (see section “Working memory training: Adaptive spatial n-back”) while receiving tDCS (ACTIVE or SHAM). At the end of the session, participants provided feedback on any perceived side effects of the stimulation. The entire session lasted approximately 45 min and was repeated for five consecutive days.

Participants returned to the lab for the outcome assessment (same tasks as at TIME 0) 48 h after the last training session (TIME 6—POST). At the end of the session, participants were debriefed on the experiment and were asked to guess their assigned stimulation group (ACTIVE or SHAM) to confirm the efficacy of the blinding procedure.

### Data analysis

Data analysis was performed using JASP (JASP Team, 2020), and R (R Core Team, 2020).

We used parametric *t*-tests to compare groups at baseline with Hedges effect sizes (*H*_*g*_) to account for small sample sizes (Hedges, 1981).

To evaluate training gains, we performed a 3-way mixed analysis of co-variance (between subject factors: STIMULATION (ACTIVE, SHAM) and AGE (YO, OO); within-subject factor: SESSION (TIME1, TIME2, TIME3, TIME4, TIME5); covariate: WMC score at baseline), on the dependent training variable [the average “n” level reached during a session ($\bar{n}$)]. Significant interactions were followed up with a simple effects analysis within each age range.

To evaluate outcome performance, we used a 3-way mixed analysis of variance [between subject factors: STIMULATION (ACTIVE, SHAM) and AGE (YO, OO); within-subject factor: SESSION (TIME0, TIME6)] to analyze the effect of the interaction between age, training, and stimulation on the composite outcome measure (WMC score). Effect sizes were quantified with partial eta square ($\eta_{p}^{2}$). We used Spearman’s ρ to quantify correlations. To test for non-random associations between categorical variables Fisher’s exact test was used, which is suitable for small sample sizes.

## Results

### Baseline differences and blinding

Tabulated statistics and descriptive data are presented in Supplementary material. Participants in the ACTIVE and SHAM groups showed no significant differences in age, years of education, MoCA scores, sleepiness, overall quality of life, depression, and familiarity with technology, either within AGE level or overall (all *p*s > 0.05). Fisher’s exact test showed no significant association between the assigned condition and the self-reported perceived stimulation, indicating that subjects were blind to the stimulation group (*p* = 0.673) (see Supplementary material for reported side effects). We also found no systematic differences in baseline WMC scores between ACTIVE and SHAM groups, within each AGE level. Thus, there were no significant differences between groups at baseline to bias the analysis. However, overall the YO and OO group differed in their WMC scores with OO having lower scores than YO [OO: −0.240 ± 0.652, YO: 0.207 ± 0.467; *t*_(26)_ = 2.108, *p* = 0.045, Hg = 0.775]. Statistics and descriptive data for the two age groups are reported in Table 2.

**TABLE 2:** Demographic and baseline characteristics of the sample divided by AGE, for each STIMULATION group.

YO	ACTIVE	SHAM	*t*(13)	*P*	Hedges’ g
N	7	8	–	–	–
Age (years)	62.71 ± 4.85	64.25 ± 4.59	0.629	0.540	0.307
Gender (F/M)^†^	4/3	6/2	–	–	–
Years of education	18.14 ± 7.69	15.00 ± 2.77	1.083	0.299	0.527
Handedness (L/R)^†⁣†^	2/5	3/5	–	–	–
WMC score	0.12 ± 0.46	0.28 ± 0.49	0.641	0.533	0.312

^†^Fisher’s exact test *p* = 0.608.					
^†⁣†^Fisher’s exact test *p* = 1.000.					

**OO**	**ACTIVE**	**SHAM**	**t(11)**	***P***	**Hedges’ g**

N	7	6	–	–	–
Age (years)	72.29 ± 2.63	73.83 ± 2.32	1.117	0.288	0.578
Gender (F/M)^†^	3/4	1/5	–	–	–
Years of education	17.14 ± 4.56	16.00 ± 6.90	0.358	0.727	0.185
Handedness (L/R)^†⁣†^	0/7	1/5	–	–	–
WMC score	−0.27 ± 0.62	−0.20 ± 0.66	0.188	0.854	0.097

^†^Fisher’s exact test *p* = 0.559.

^†⁣†^Fisher’s exact test *p* = 0.462.

For each subsample and variable, we report the count *N* and the average score, together with its standard deviation, Welch’s t statistics, corresponding *p*-value and effect size (Hedges’ g) from an independent *t*-test between ACTIVE and SHAM.

### Training gains

Following previous findings in young (Assecondi et al., 2021) and older adults (Krebs et al., 2021), we hypothesized that individuals entering the training regimen with lower working memory capacity would benefit more from the intervention.

**FIGURE 3:**
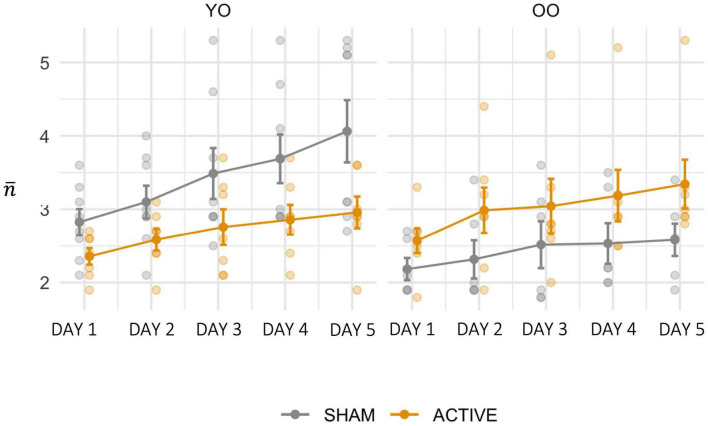
Training performance ($\bar{n}$) for each training day, for the SHAM and ACTIVE groups, within each AGE level. Error bars represent the standard error of the mean.

Performance across groups is depicted in Figure 3. The effect of the baseline WMC scores covariate was significant [*F*_(1, 23)_ = 10.736, *p* = 0.003, $\eta_{p}^{2}$ = 0.318], with higher scores corresponding to enhanced training performance. There was also a significant main effect of SESSION [*F*_(4_, _92)_ = 11.276, *p* < 0.001, $\eta_{p}^{2}$ = 0.329] and a significant STIMULATION × AGE interaction [*F*_(1, 23)_ = 10.846, *p* = 0.003, $\eta_{p}^{2}$ = 0.320], after controlling for initial working memory. Simple effects analyses revealed that ACTIVE stimulation was more effective than SHAM at increasing performance (*p* = 0.027) in the OO group, while the opposite was found in the YO, with a larger increase in performance found in the SHAM condition compared to ACTIVE (*p* = 0.016).

To summarize, our finding supports the hypothesis that the benefit of combined training regimens is inversely proportional to initial working memory capacity. Importantly, old-old individuals with lower capacity (OO) benefit the most in training gains from the combination of training and brain stimulation relative to cognitive training alone, with this advantage evident from DAY1.

### Outcome tasks

To assess the overall impact of combined tDCS and working memory training on working memory, we assessed changes in WMC score after five consecutive days of training (POST). Analysis of individual tasks is reported in Supplementary material.

The analysis of the change in WMC score revealed a significant main effect of SESSION [*F*_(1, 24)_ = 45.502, *p* < 0.001, $\eta_{p}^{2}$ = 0.655] and of AGE [*F*_(1, 24)_ = 4.665, *p* = 0.041, $\eta_{p}^{2}$ = 0.163]. Follow-up analysis showed that WMC scores were significantly higher at post than at pretest, and YO scores were significantly higher than OO scores, regardless of the stimulation. To summarize, overall working memory evaluated 2 days after the end of 5 days of practice, increased in both age groups, with overall higher scores in young-old, but this increase was not modulated by stimulation. This result could be ascribed to an overall benefit of the training or due to practice effects or both.

### Strategy use

In a previous study with young adults, we found that strategy plays an important role in combined working memory training and stimulation regimens (Assecondi, 2021). Therefore, we collected self-reports of strategy use at the end of training by asking participants two questions, with answers ranging on a scale from 1 to 10: (1) if they used a strategy; and, if so, (2) how efficient they felt the strategy was in improving their performance.

Overall, we found no significant association between STIMULATION groups and frequency of strategy use, meaning that individuals in the ACTIVE and SHAM groups were equally likely to develop a strategy. We found a difference between the perceived efficacy of the strategy between the ACTIVE and SHAM group, however, (t_Welch_ = 2.239, *p* = 0.037, H_g_ = 0.889), with the SHAM group perceiving the strategy to be more efficient (ACTIVE: 7.12 ± 0.41; SHAM: 8.22 ± 0.28). However, the rating of the strategy’s effectiveness did not correlate with training gains (Spearman’s ρ = −0.053, *p* = 0.819, *N* = 20).

When looking at the frequency of strategy use separately for YO and OO individuals (see Supplementary material for details), however, we found a significant association between STIMULATION and strategy use for OO individuals only (Fisher’s exact *p* = 0.005), meaning that OO individuals receiving active stimulation were more likely to use a strategy than those not receiving active stimulation.

## Discussion

The purpose of the present study was to investigate the effectiveness of improving working memory in older individuals by combining working memory training and transcranial direct current stimulation. We employed a 5-day training regimen, where participants completed 20 min of combined working memory training and tDCS (SHAM or ACTIVE) on five consecutive weekdays. Two days after the end of the intervention, we evaluated training effects on an overall composite WMC score. We found that, whereas all individuals improved their performance during training, tDCS concurrent with working memory training selectively benefitted old-old individuals with lower working memory capacity. We also found that performance during training was significantly better when there was no stimulation in young-old adults, who showed higher working memory scores.

Non-invasive brain stimulation, in our case tDCS, is emerging as a “state-dependent” intervention, with its effectiveness modulated by the pre-existing individual’s cortical excitation (Krause et al., 2013; Hsu et al., 2014; Krause and Cohen Kadosh, 2014; Esposito et al., 2021, 2022). The relation between excitation and performance has been proposed as a left-skewed inverted U-shaped curve, with a maximum point corresponding to optimal performance. Cognitive functioning within the curve is optimal when there is a balance between inhibition and excitation to allow sufficient flexibility for acquiring new skills (plasticity) but equally sufficient stability to resist noise (e.g., distractions) and to maintain new information (Krause et al., 2013; Krause and Cohen Kadosh, 2014).

Aging is associated with a weaker cortical excitability (Rossini et al., 1992; Pitcher et al., 2003; Oliviero et al., 2006; Ferreri et al., 2017). Importantly, however, engaging in a task increases cortical excitability. It is illustrative to compare a young individual with an older individual. The former will have higher WMC scores, stronger cortical excitability, and resources to engage with the task; the latter will likely have lower WMC scores, weaker cortical excitability, hence less cortical activation, and fewer resources available for the task. The younger individual is likely closer to its optimal performance point (maximum on the u-shaped curve). When receiving tDCS, their cortical excitability will increase, potentially pushing this individual over their optimal performance point, resulting in worse performance. On the other hand, the older adult is likely further away from their optimal performance point; therefore, receiving tDCS will only push them closer to their maximum of the u-shaped curve, resulting in better performance. This model agrees with the inverted effect of tDCS we found in young-old and old-old adults. Our findings also agree with both the cognitive training and the tDCS literature, showing that training-related changes in performance correlate with initial WMC scores. Whereas cognitive training alone seems more effective in young-old adults (Berryhill and Jones, 2012; Foster et al., 2017), the benefits of tDCS, when added to cognitive training, seem to be specific to old-old adults (Tseng et al., 2012; Gözenman and Berryhill, 2016; Assecondi et al., 2021; Krebs et al., 2021). Identifying the neural mechanism that explains this selective tDCS benefit warrants further research (Tseng et al., 2012).

A working memory training regimen of 5 days is relatively short with respect to typical cognitive training studies. Despite this short training period, we also assessed overall working memory improvements, as measured *via* a composite working memory score. This composite WMC score includes tasks that share information processing components and engage similar neural circuitry (Dahlin et al., 2008). Research has shown that change in performance after training can be due to either a change in capacity or a change in efficiency, depending on available capacity (von Bastian and Oberauer, 2014). Whereas working memory scores improved overall, we found no significant differences in composite score due to stimulation. It is worth noting that the WMC score is calculated from different types of tasks, which could be considered to range from near- to far- transfer tasks. Lack of transfer effects has been reported before (Waris et al., 2015; Soveri et al., 2017a,b) and, in our study, possibly exacerbated by the short duration of the training program.

We found that old-old individuals receiving active stimulation were more likely to use a strategy than those not receiving active stimulation. We can hypothesize that tDCS to the frontal area may facilitate executive functions, and therefore, promote strategy use. We acknowledge though that this statement is highly speculative, particularly considering our sample size, and further research is needed to test this hypothesis.

Regarding limitations, we acknowledge that our sample size was small (Button et al., 2013), however, we took several steps to support our findings, including implementing a model that accounts for subject variability (in age and initial WMC score). We did not evaluate cognitive function after a period of no contact; we, therefore, cannot say if there was a modulatory effect in maintaining the training gain for a more extended period. Although it will be useful to replicate our results in a larger sample size, we note that we did replicate results found in a young adult population and that our findings are consistent with the literature (Assecondi, 2021; Krebs et al., 2021).

To conclude, we found that tDCS during working memory training selectively benefits old-old adults with lower initial working memory capacity; importantly, we also show that the benefit of training in older adults is predicted by baseline working memory capacity (Au et al., 2022). The combined intervention appears to be a viable method to improve working memory in young and older adults, the latter being crucial given the large cohort of older adults experiencing both normal and abnormal (e.g., dementia) cognitive decline.

Further investigation of transfer benefits is needed in longer-term training studies along with an investigation of the role of strategy development. Finally, it will be essential to investigate the neurophysiological correlates of training-related changes, e.g., using EEG, to understand if these are caused by an improvement in a specific working memory component, such as in capacity or general strategy use. Our research adds to the body of literature that stresses the importance of interindividual differences to explain the variability in the outcome of tDCS studies. Importantly, it shows how this variability can be exploited to maximize the outcome of cognitive training interventions. A better understanding of the mechanism underlying successful training and transfer will likely benefit the design of subject-specific training and training regimens in general.

## Data availability statement

The raw data supporting the conclusions of this article will be made available by the authors, without undue reservation.

## Ethics statement

The studies involving human participants were reviewed and approved by Ethics Committee of the University of Birmingham. The patients/participants provided their written informed consent to participate in this study.

## Author contributions

SA: conceptualization, methodology, validation, data collection and curation, formal analysis, investigation, writing—original draft and review and editing, and funding acquisition. RH: data collection and writing—review and editing. JK: methodology and data collection and analyses. GE: conceptualization, methodology, resources, writing—review and editing, and funding acquisition. KS: conceptualization, methodology, resources, supervision, writing—review and editing, and funding acquisition. All authors contributed to the article and approved the submitted version.

## Footnotes

1 DVz = (DV-μ)/σ, where DVz is the standardized dependent variable, DV is the original dependent variable, μ and σ are the mean and standard deviation of the DV over the entire sample at baseline (28 participants).

2 One participant let 45 days pass between baseline testing and the first training session because of unforeseen family circumstances conflicting with scheduling. The participant was included in the analysis because we did not expect a significant change in their cognitive function over 45 days. As an additional check, repeating the analysis of the training data without that participant did not change the significance of the results.
