# Supplementary material for: Older adults with lower working memory capacity benefit from transcranial direct current stimulation when combined with working memory training: A preliminary study
Source: Front Aging Neurosci. 2022 Oct 10;14:1009262. doi: 10.3389/fnagi.2022.1009262 (PMC9589058; doi:10.3389/fnagi.2022.1009262)
Supplement: Supplementary file 1 [file Data_Sheet_1.docx]

Supplementary Material

[1 Current density modelling 2](#_Toc115367407)

[2 Tasks description 3](#_Toc115367408)

[2.1 Practice effects: non-adaptive spatial n-Back (SNBACK) 3](#_Toc115367409)

[2.2 Near-transfer effects: Visual n-back (VNBACK) 3](#_Toc115367410)

[2.3 Near-transfer effects: Change detection task 3](#_Toc115367411)

[2.4 Far-transfer effects: Digit Span task 4](#_Toc115367412)

[3 Statistical analysis of individual tasks 5](#_Toc115367413)

[3.1 Training task 5](#_Toc115367414)

[3.2 Outcome tasks 6](#_Toc115367415)

[4 Baseline characteristics 8](#_Toc115367416)

[4.1 Demographic and baseline characteristics 8](#_Toc115367417)

[4.2 Baseline cognitive state and lifestyle 9](#_Toc115367418)

[5 Mood and attitude throughout the training 11](#_Toc115367419)

[6 Possible adverse effects of brain stimulation 12](#_Toc115367420)

[7 Strategy use 13](#_Toc115367421)

# Current density modelling

Right, frontal, and left view of the simulated electric field (normal component (En)), above and current density (below) with the tDCS configuration used in the study (F4 active electrode, Fp1 return electrode, 2mA and circular electrodes with a 3.14 cm2 area). The simulated electric field and current density distribution were obtained with Simnibs (Thielscher et al., 2015). A positive value for the component of the electric field normal to the cortical surface means the electric field normal component is pointing into the cortex, and such a field would be excitatory. On the other hand, an electric field pointing out of the cortex (negative normal component) would be inhibitory.

| **ELECTRIC FIELD (V/m)** | | |
| --- | --- | --- |
| RIGHT VIEW | FRONTAL VIEW | LEFT VIEW |
| 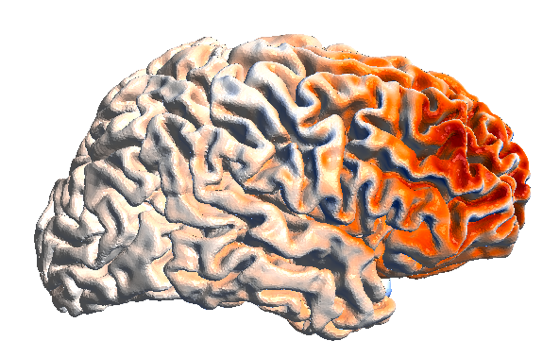 | 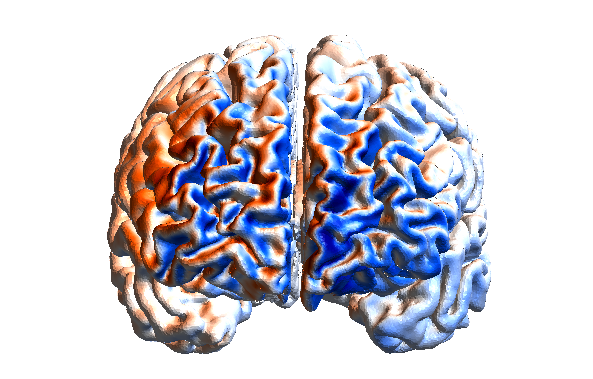 | 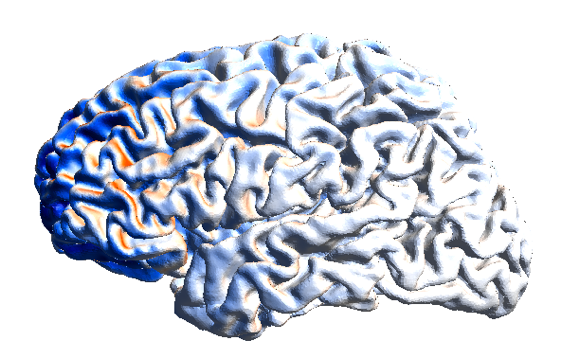 |
| 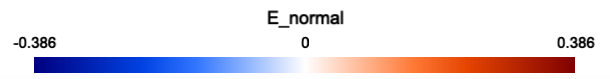 | | |

| **CURRENT DENSITY (A/m^2^)** | | |
| --- | --- | --- |
| RIGHT VIEW | FRONTAL VIEW | LEFT VIEW |
| 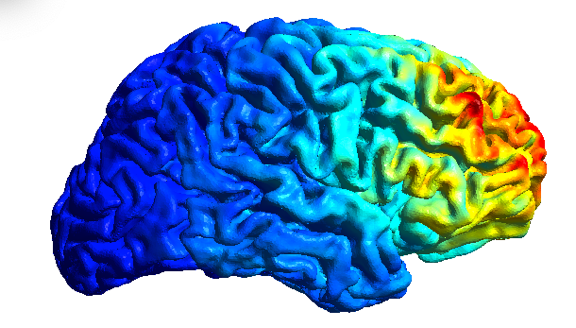 | 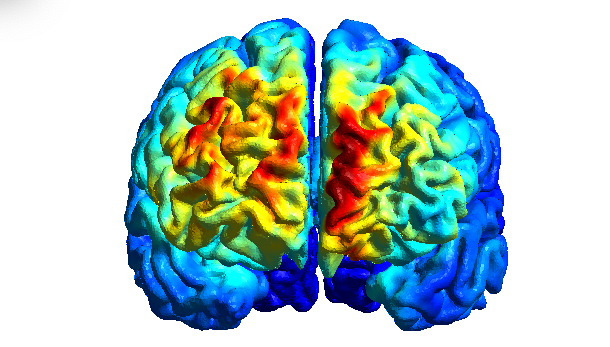 | 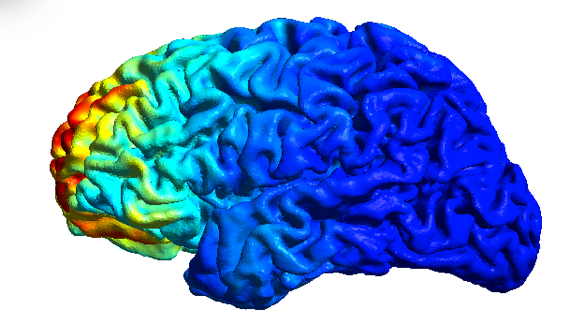 |
| 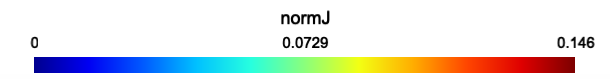 | | |

# Tasks description

## Practice effects: non-adaptive spatial n-Back (SNBACK)

A non-adaptive version of the N-IGMA task was used to assess training-related gains. Parameters of the task were the same as the training task (section 2.3, main manuscript, and Figure 1 panel A here), but in this case participants completed only 6 blocks of ‘n’= 1 and 6 blocks of ‘n’=2. To compute the dependent variables we extracted the d-prime (d’) for each ‘n’, as follows (Macmillan and Creelman, 2005):

$$d^{'}=z\left( H \right)-z\left( FA \right)$$

where H, FA are the fraction of hits and false alarms with respect to match and no-match trials.


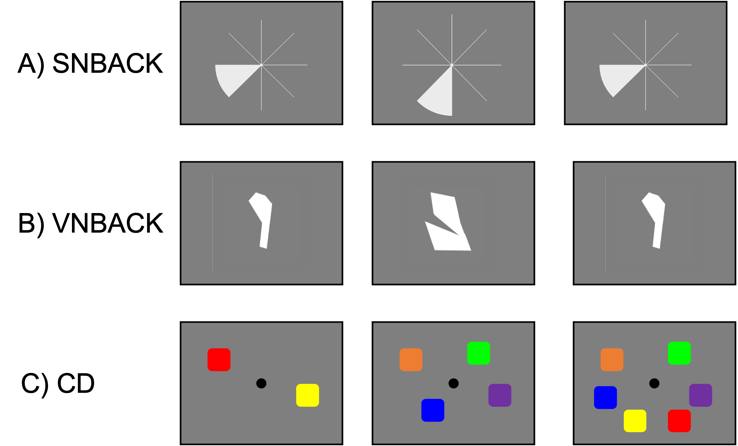


Figure 1 Examples of the stimuli used in the study, for three of the four outcome tasks: A) spatial n-back (SNBACK), a wedge on a circular grid; B) visual n-back (VNBACK), irregular shapes; C) Change detection (CD), 2,4, or 6 coloured squares.

## Near-transfer effects: Visual n-back (VNBACK)

To evaluate near-transfer effects, we used two working memory tasks: a visual n-back task (Figure 1 panel B) and a change detection task. In the visual n-back task, participants were presented with sequential non-geometrical random shapes^^[[1]](#footnote-1)^^ (Vanderplas & Garvin, 1959) in the center of the screen for 0.5 sec, followed by a 2.5 sec maintenance interval (fixation on a grey background) and asked to respond to changes (different shape than ‘n’ before) by pressing the left (‘match’) or the right (‘no match’) arrow on the keyboard. The shapes were chosen to prevent participants labelling them, thus avoiding the use of verbal memory. Each session consisted of 6 blocks of a ‘n’=1 and 6 blocks of a ‘n’=2, with 20+N stimuli (6 matches) per block. Again, d-prime (d’) was used as dependent variables.

## Near-transfer effects: Change detection task

In the change detection task (Luck and Vogel, 1997), participants were presented with two arrays, containing a specified number of items (squares) separated by a short delay (Figure 1 panel C). The memory array contained 2, 4, or 6 distinct colored squares^^[[2]](#footnote-2)^^ and was presented for 0.5 sec. After a delay (1 sec), a second (test) array was presented, containing only one square, which remained on the screen until participants responded whether the square in the test array was the same color (50% of trials, match, left arrow) or a different color (50% of trials, no match, right arrow) than the square in the same position on the memory array. Participants completed 4 blocks of 48 trials each, randomly presented. In addition to d-prime (d’) for each set size, for the CD task, we also derived the single probe working memory capacity index (K, (Cowan, 2001; Rouder et al., 2011)), averaged across set size $s_{i=1..I}$, as follows:

$$K=\frac{1}{I}\sum_{i} s_{i}*[z\left( H \right)-z\left( \mathrm{FA} \right)]$$

## Far-transfer effects: Digit Span task

Participants were presented with a series of digits delivered via headphones at a rate of one stimulus per second and requested to use the keyboard to respond to the sequence as they heard it (in the forward version) or backward (in the backwards version). Each trial consisted of two sequences of the same span. Starting from a sequence of length two, two consecutive errors would stop the task, whereas with only one error the task would proceed to the next trial, increasing span by one. No time limit was placed on the response (Kreutzer et al., 2011; Wechsler Adult Intelligence Scale--Fourth Edition - PsycNET). Dependent variable was the maximum span, i.e., the length of the longest string of digits repeated without error over two consecutive trials.

# Statistical analysis of individual tasks

## Training task

3-way mixed analysis of co-variance (between subject: STIMULATION (ACTIVE, SHAM) x AGE (YO, OO); within-subject: SESSION (DAY1, DAY2, DAY3, DAY4, DAY5); covariate: WMC score at baseline), of the dependent variable (the average ‘n’ level reached during a session ($\bar{n}$)). Significance is marked in red.

| Training task ASNBACK | | | | |
| --- | --- | --- | --- | --- |
| **EFFECT** | **df** | **F** | **p-value** | **η²_p_** |
| SESSION | 4,92 | 11.276 | < .001 | 0.329 |
| SESSION * STIMULATION | 4,92 | 0.282 | 0.793 | 0.012 |
| SESSION * AGE | 4,92 | 0.567 | 0.600 | 0.024 |
| SESSION * WMC score | 4,92 | 1.741 | 0.179 | 0.070 |
| SESSION * STIMULATION * AGE | 4,92 | 1.154 | 0.329 | 0.048 |
| STIMULATION | 1,23 | 0.004 | 0.948 | 0.000 |
| AGE | 1,23 | 0.172 | 0.682 | 0.007 |
| WMC score | 1,23 | 10.736 | 0.003 | 0.318 |
| STIMULATION * AGE | 1,23 | 10.846 | 0.003 | 0.320 |
| ++ N_mu ~ Session * Stimulation * Age * WMCS | |  |  |  |
|  |  |  |  |  |
| **SIMPLE MAIN EFFECTS OF STIMULATION WITHIN AGE** | | |  |  |
| **AGE** | **df** | **F** | **p-value** |  |
| OO | 1 | 5.556 | 0.027 |  |
| YO | 1 | 6.736 | 0.016 |  |

## Outcome tasks

3-way mixed analysis of co-variance (between subject: STIMULATION (ACTIVE, SHAM) x AGE (YO, OO); within-subject: SESSION (PRE, POST); covariate: WMC score at baseline), of the dependent variable considered in each task. Significance is marked in red.

| **OUTCOME TASKS** | | | | | |
| --- | --- | --- | --- | --- | --- |
| **SNBACK** | **EFFECT** | **df** | **F** | **p-value** | **η²_p_** |
|  | SESSION | 1,23 | 86.583 | < .001 | 0.790 |
|  | SESSION * STIMULATION | 1,23 | 0.001 | 0.973 | 0.000 |
|  | SESSION * AGE | 1,23 | 3.489 | 0.075 | 0.132 |
|  | SESSION * WMC score | 1,23 | 2.021 | 0.169 | 0.081 |
|  | SESSION * STIMULATION * AGE | 1,23 | 4.079 | 0.055 | 0.151 |
|  | STIMULATION | 1,23 | 1.836 | 0.189 | 0.074 |
|  | AGE | 1,23 | 1.377 | 0.253 | 0.056 |
|  | WMC score | 1,23 | 36.694 | < .001 | 0.615 |
|  | STIMULATION * AGE | 1,23 | 10.505 | 0.004 | 0.314 |
|  | D ~ Session * Stimulation * Age * WMCS | | | | |
|  | **SIMPLE MAIN EFFECTS OF STIMULATION WITHIN AGE** | | |  |  |
|  | **AGE** | **df** | **F** | **p-value** |  |
|  | OO | 1 | 9.987 | 0.004 |  |
|  | YO | 1 | 2.07 | 0.164 |  |
|  |  |  |  |  |  |
| **VNBACK** | **EFFECT** | **df** | **F** | **p-value** | **η²_p_** |
|  | SESSION | 1,23 | 22.990 | < .001 | 0.500 |
|  | SESSION * STIMULATION | 1,23 | 0.060 | 0.808 | 0.003 |
|  | SESSION * AGE | 1,23 | 0.549 | 0.466 | 0.023 |
|  | SESSION * WMC score | 1,23 | 2.106 | 0.16 | 0.084 |
|  | SESSION * STIMULATION * AGE | 1,23 | 5.208 | 0.032 | 0.185 |
|  | STIMULATION | 1,23 | 0.164 | 0.690 | 0.007 |
|  | AGE | 1,23 | 0.585 | 0.452 | 0.025 |
|  | WMC score | 1,23 | 16.817 | < .001 | 0.422 |
|  | STIMULATION * AGE | 1,23 | 1.642 | 0.213 | 0.067 |
|  | D ~ Session * Stimulation * Age * WMCS | | | | |
|  | **SIMPLE MAIN EFFECTS OF STIMULATION WITHIN AGE** | | |  |  |
|  | **AGE** | **df** | **F** | **p-value** |  |
|  | OO | 1 | 0.200 | 0.664 |  |
|  | YO | 1 | 3.455 | 0.088 |  |
|  |  |  |  |  |  |
|  |  |  |  |  |  |
|  |  |  |  |  |  |
|  |  |  |  |  |  |
|  |  |  |  |  |  |
|  |  |  |  |  |  |
|  |  |  |  |  |  |
| **CD** | **EFFECT** | **df** | **F** | **p-value** | **η²_p_** |
|  | SESSION | 1,23 | 12.274 | 0.002 | 0.348 |
|  | SESSION * STIMULATION | 1,23 | 1.025 | 0.322 | 0.043 |
|  | SESSION * AGE | 1,23 | 0.393 | 0.537 | 0.017 |
|  | SESSION * WMC score | 1,23 | 0.194 | 0.663 | 0.008 |
|  | SESSION * STIMULATION * AGE | 1,23 | 0.174 | 0.681 | 0.007 |
|  | STIMULATION | 1,23 | 0.905 | 0.351 | 0.038 |
|  | AGE | 1,23 | 0.000 | 1.000 | 0.000 |
|  | WMC score | 1,23 | 5.843 | 0.024 | 0.203 |
|  | STIMULATION * AGE | 1,23 | 0.262 | 0.614 | 0.011 |
|  | D ~ Session * Stimulation * Age * WMCS | | | | |
|  |  |  |  |  |  |
| BACKWARD DIGIT SPAN | **EFFECT** | **df** | **F** | **p-value** | **η²_p_** |
|  | SESSION | 1,23 | 2.252 | 0.147 | 0.089 |
|  | SESSION * STIMULATION | 1,23 | 0.728 | 0.402 | 0.031 |
|  | SESSION * AGE | 1,23 | 0.018 | 0.895 | 0.001 |
|  | SESSION * WMC score | 1,23 | 1.015 | 0.324 | 0.042 |
|  | SESSION * STIMULATION * AGE | 1,23 | 0.600 | 0.446 | 0.025 |
|  | STIMULATION | 1,23 | 0.755 | 0.394 | 0.032 |
|  | AGE | 1,23 | 0.197 | 0.661 | 0.008 |
|  | WMC score | 1,23 | 20.192 | < .001 | 0.467 |
|  | STIMULATION * AGE | 1,23 | 0.230 | 0.636 | 0.010 |
|  | D ~ Session * Stimulation * Age * WMCS | | | | |
|  |  |  |  |  |  |
| FORWARD DIGIT SPAN | **EFFECT** | **df** | **F** | **p-value** | **η²_p_** |
|  | SESSION | 1,23 | 0.847 | 0.367 | 0.036 |
|  | SESSION * STIMULATION | 1,23 | 0.045 | 0.834 | 0.002 |
|  | SESSION * AGE | 1,23 | 0.095 | 0.760 | 0.004 |
|  | SESSION * WMC score | 1,23 | 0.373 | 0.547 | 0.016 |
|  | SESSION * STIMULATION * AGE | 1,23 | 0.263 | 0.613 | 0.011 |
|  | STIMULATION | 1,23 | 0.892 | 0.355 | 0.037 |
|  | AGE | 1,23 | 0.001 | 0.982 | 0.000 |
|  | WMC score | 1,23 | 2.757 | 0.110 | 0.107 |
|  | STIMULATION * AGE | 1,23 | 1.358 | 0.256 | 0.056 |
|  | D ~ Session * Stimulation * Age * WMCS | | | | |
|  |  |  |  |  |  |

# Baseline characteristics

## Demographic and baseline characteristics

Demographic characteristics and baseline scores are reported in Table I. Statistical tests showed that the four groups did not differ in age, years of education, motivation, mood attitude or baseline performance (all ps > 0.05). A chi-square test of independence was performed to examine the relation between groups (ACTIVE, SHAM) and AGE. The relation between these variables was not significant (X^2^_(df=1, N=28)_ = 0.144, p= 0.705), indicating that groups were equally likely to contain YO and OO individuals. Fisher’s exact test of independence showed no significant relation between STIMULATION and gender or handedness.

| Table I Demographic and baseline characteristics of the overall sample, and of the sample divided by AGE, for each STIMULATION group. For each subsample and variable, we report the count N and the average score, together with its standard deviation, Welch’s t statistics, corresponding p-value and effect size ( $\mathbf{Hedges}^{\mathbf{'}}\boldsymbol{g}$) from an independent t-test between ACTIVE and SHAM.   \| OVERALL \| ACTIVE \| SHAM \| t(26) \| p \| Hedges' g \| \| \| --- \| --- \| --- \| --- \| --- \| --- \| --- \| \| N \| 14 \| 14 \| -- \| -- \| -- \| \| \| Age (years) \| 67.50 ± 6.22 \| 68.36 ± 6.13 \| 0.367 \| 0.717 \| 0.135 \| \| \| Gender (F/M) † \| 7 / 7 \| 7 / 7 \| -- \| -- \| -- \| \| \| Years of education \| 17.64 ± 6.10 \| 15.45 ± 4.77 \| 1.071 \| 0.294 \| 0.393 \| \| \| Handedness (L/R) †† \| 2 / 12 \| 4 / 10 \| -- \| -- \| -- \| \| \| WMC score \| -0.08 ± 0.60 \| 0.07 ± 0.60 \| 0.657 \| 0.517 \| 0.241 \| \| \| †Fisher’s exact test p = 1.000 ††Fisher’s exact test p = 0.648 \| \| \| \| \| \| |
| --- | --- | --- | --- | --- | --- | --- | --- | --- | --- | --- | --- | --- | --- | --- | --- | --- | --- | --- | --- | --- | --- | --- | --- | --- | --- | --- | --- | --- | --- | --- | --- | --- | --- | --- | --- | --- | --- | --- | --- | --- | --- | --- | --- | --- | --- | --- | --- | --- | --- | --- | --- | --- | --- | --- | --- |
| \| YO \| ACTIVE \| SHAM \| t(13) \| p \| Hedges' g \| \| --- \| --- \| --- \| --- \| --- \| --- \| \| N \| 7 \| 8 \| -- \| -- \| -- \| \| Age (years) \| 62.71 ± 4.85 \| 64.25 ± 4.59 \| 0.629 \| 0.540 \| 0.307 \| \| Gender (F/M)† \| 4 / 3 \| 6 / 2 \| -- \| -- \| -- \| \| Years of education \| 18.14 ± 7.69 \| 15.00 ± 2.77 \| 1.083 \| 0.299 \| 0.527 \| \| Handedness (L/R)†† \| 2 / 5 \| 3 / 5 \| -- \| -- \| -- \| \| WMC score \| 0.12 ± 0.46 \| 0.28 ± 0.49 \| 0.641 \| 0.533 \| 0.312 \| \| †Fisher’s exact test p = 0.608  ††Fisher’s exact test p = 1.000 \| |
| \| OO \| ACTIVE \| SHAM \| t(11) \| p \| Hedges' g \| \| --- \| --- \| --- \| --- \| --- \| --- \| \| N \| 7 \| 6 \| -- \| -- \| -- \| \| Age (years) \| 72.29 ± 2.63 \| 73.83 ± 2.32 \| 1.117 \| 0.288 \| 0.578 \| \| Gender (F/M) † \| 3 / 4 \| 1 / 5 \| -- \| -- \| -- \| \| Years of education \| 17.14 ± 4.56 \| 16.00 ± 6.90 \| 0.358 \| 0.727 \| 0.185 \| \| Handedness (L/R)†† \| 0 / 7 \| 1 / 5 \| -- \| -- \| -- \| \| WMC score \| -0.27 ± 0.62 \| -0.20 ± 0.66 \| 0.188 \| 0.854 \| 0.097 \| \| †Fisher’s exact test p = 0.559  ††Fisher’s exact test p = 0.462 \| \| \| \| \| \| |

## Baseline cognitive state and lifestyle

Baseline cognitive status, emotional symptoms and sleepiness are reported in Table II. We compared cognitive functions, habitual sleepiness (ESS), familiarity with technology (FWT), Quality of life (QoL), anxiety, and depression, as measured by the Hospital anxiety and depression scale, in the whole sample and in the YO and OO subsamples, between ACTIVE and SHAM. We found no significant difference between ACTIVE and SHAM overall, or within AGE groups.

| Table II Cognitive and lifestyle measures at baseline of the overall sample, and of the sample divided by AGE, for each STIMULATION group. For each subsample and variable, we report the count N and the average score, together with its standard deviation, Welch’s t statistics, corresponding p-value and effect size ($\mathbf{Hedges}^{\mathbf{'}}\boldsymbol{g}$) from an independent t-test between ACTIVE and SHAM. MoCA = Montreal Cognitive Assessment, ESS= Epworth Sleepiness Scale, FWT = Familiarity with technology; QoL = Quality of Life; ANXIETY and DEPRESSION as measured by the Hospital Anxiety and Depression Scale. Significant findings are marked with *.     \|  \|  \| **ACTIVE** \| **SHAM** \| **df** \| **t** \| **p** \| **Hedges’ g** \| \| --- \| --- \| --- \| --- \| --- \| --- \| --- \| --- \| \| **OVERALL** \| **MOCA** \| 28.71 ± 1.27 \| 28.43 ± 1.45 \| 26 \| 0.555 \| 0.584 \| 0.204 \| \| **ESS** \| 4.64 ± 2.62 \| 5.14 ± 3.13 \| 26 \| 0.458 \| 0.651 \| 0.168 \| \| **FWT** \| 21.79 ± 5.59 \| 20.43 ± 5.02 \| 26 \| 0.676 \| 0.505 \| 0.248 \| \| **QoL** \| 58.13 ± 5.08 \| 57.47 ± 5.77 \| 26 \| 0.321 \| 0.751 \| 0.118 \| \| **ANXIETY** \| 3.79 ± 2.52 \| 4.71 ± 2.52 \| 26 \| 0.975 \| 0.339 \| 0.358 \| \| **DEPRESSION** \| 2.86 ± 2.98 \| 2.79 ± 2.78 \| 26 \| 0.066 \| 0.948 \| 0.024 \| \| **OO** \| **MOCA** \| 28.57 ± 1.27 \| 27.67 ± 1.50 \| 11 \| 1.159 \| 0.274 \| 0.604 \| \| **ESS** \| 3.28 ± 1.98 \| 6.67 ± 4.18 \| 11 \| 1.815 \| 0.113 \| 0.962 \| \| **FWT** \| 21.71 ± 6.16 \| 18.00 ± 4.56 \| 11 \| 1.246 \| 0.239 \| 0.638 \| \| **QoL** \| 58.85 ± 4.86 \| 58.68 ± 7.69 \| 11 \| 0.048 \| 0.963 \| 0.025 \| \| **ANXIETY** \| 4.14 ± 2.73 \| 3.67 ± 3.01 \| 11 \| 0.297 \| 0.773 \| 0.154 \| \| **DEPRESSION** \| 1.71 ± 1.60 \| 3.33 ± 3.33 \| 11 \| 0.401 \| 0.313 \| 0.577 \| \| **YO** \| **MOCA** \| 28.85 ± 1.34 \| 29.00 ± 1.19 \| 13 \| 0.216 \| 0.833 \| 0.106 \| \| **ESS** \| 6.00 ± 2.58 \| 4.00 ± 1.51 \| 13 \| 1.797 \| 0.104 \| 0.89 \| \| **FWT** \| 21.86 ± 5.46 \| 22.25 ± 4.80 \| 13 \| 0.147 \| 0.886 \| 0.072 \| \| **QoL** \| 57.41 ± 5.57 \| 56.57 ± 4.17 \| 13 \| 0.328 \| 0.749 \| 0.161 \| \| **ANXIETY** \| 3.43 ± 2.44 \| 5.50 ± 1.93 \| 13 \| 1.807 \| 0.097 \| 0.887 \| \| **DEPRESSION** \| 4.00 ± 3.70 \| 2.37 ± 2.45 \| 13 \| 0.989 \| 0.346 \| 0.488 \|   * p < .05, ** p < .01, *** p < .001 |
| --- | --- | --- | --- | --- | --- | --- | --- | --- | --- | --- | --- | --- | --- | --- | --- | --- | --- | --- | --- | --- | --- | --- | --- | --- | --- | --- | --- | --- | --- | --- | --- | --- | --- | --- | --- | --- | --- | --- | --- | --- | --- | --- | --- | --- | --- | --- | --- | --- | --- | --- | --- | --- | --- | --- | --- | --- | --- | --- | --- | --- | --- | --- | --- | --- | --- | --- | --- | --- | --- | --- | --- | --- | --- | --- | --- | --- | --- | --- | --- | --- | --- | --- | --- | --- | --- | --- | --- | --- | --- | --- | --- | --- | --- | --- | --- | --- | --- | --- | --- | --- | --- | --- | --- | --- | --- | --- | --- | --- | --- | --- | --- | --- | --- | --- | --- | --- | --- | --- | --- | --- | --- | --- | --- | --- | --- | --- | --- | --- | --- | --- | --- | --- | --- | --- | --- | --- | --- |

| Table III Pairwise correlation (Spearman’ ρ) of cognitive performance at baseline (d’ in SNBACK, VNBACK and CD, and SPAN in DSB and DSF) for the whole sample. Significant correlations are marked with *.   \| PAIRS \| Spearman’s rho (N=28) \| p \| \| --- \| --- \| --- \| \| CD – DSF \| -0.039 \| 0.843 \| \| CD – DSB \| -0.122 \| 0.536 \| \| CD – VNBACK_D \| 0.420* \| 0.027 \| \| CD – SNBACK_D \| 0.361 \| 0.060 \| \| DSF_SPAN – DSB_SPAN \| 0.227 \| 0.245 \| \| DSF_SPAN – VNBACK_D \| 0.095 \| 0.629 \| \| DSF_SPAN – SNBACK_D \| 0.068 \| 0.731 \| \| DSB_SPAN – VNBACK_D \| 0.324 \| 0.093 \| \| DSB_SPAN – SNBACK_D \| 0.484** \| 0.009 \| \| VNBACK_D – SNBACK_D \| 0.634 *** \| < .001 \| \|  \| \| \| \| * p < .05, ** p < .01, *** p < .001 \| \| \| |
| --- | --- | --- | --- | --- | --- | --- | --- | --- | --- | --- | --- | --- | --- | --- | --- | --- | --- | --- | --- | --- | --- | --- | --- | --- | --- | --- | --- | --- | --- | --- | --- | --- | --- | --- | --- | --- | --- | --- | --- |

# Mood and attitude throughout the training

Table IV Output of a 3-way mixed ANOVA (between subjects: STIMULATION: ACTIVE, CONTROL x AGE: YO,OO; within-subject: SESSION: DAY1, DAY2, DAY3, DAY4, DAY5 for each day of combined training and stimulation) on attitude and expectation.

| **ALERTNESS** |  | **df** | **F** | **p** | **η²_p_** |
| --- | --- | --- | --- | --- | --- |
|  | SESSION | 4,24 | 0.299 | 0.878 | 0.012 |
|  | SESSION * STIMULATION | 4,24 | 0.458 | 0.767 | 0.019 |
|  | SESSION * AGE | 4,24 | 0.745 | 0.563 | 0.030 |
|  | SESSION * STIMULATION * AGE | 4,24 | 2.018 | 0.098 | 0.078 |
|  | STIMULATION | 1,24 | 0.029 | 0.866 | 0.001 |
|  | AGE | 1,24 | 0.290 | 0.595 | 0.012 |
|  | STIMULATION * AGE | 1,24 | 0.022 | 0.884 | 0.001 |
| **MOTIVATION** |  | **df** | **F** | **p** | **η²_p_** |
|  | SESSION | 4,24 | 0.308 | 0.822 | 0.013 |
|  | SESSION * STIMULATION | 4,24 | 0.343 | 0.797 | 0.014 |
|  | SESSION * AGE | 4,24 | 0.647 | 0.589 | 0.026 |
|  | SESSION * STIMULATION * AGE | 4,24 | 0.254 | 0.860 | 0.010 |
|  | STIMULATION | 1,24 | 0.477 | 0.496 | 0.020 |
|  | AGE | 1,24 | 0.075 | 0.787 | 0.003 |
|  | STIMULATION * AGE | 1,24 | 0.219 | 0.644 | 0.009 |
| **SADNESS** |  | **df** | **F** | **p** | **η²_p_** |
|  | SESSION | 4,24 | 0.682 | 0.527 | 0.028 |
|  | SESSION * STIMULATION | 4,24 | 0.331 | 0.744 | 0.014 |
|  | SESSION * AGE | 4,24 | 1.641 | 0.201 | 0.064 |
|  | SESSION * STIMULATION * AGE | 4,24 | 0.066 | 0.951 | 0.003 |
|  | STIMULATION | 1,24 | 0.616 | 0.440 | 0.025 |
|  | AGE | 1,24 | 0.961 | 0.337 | 0.038 |
|  | STIMULATION * AGE | 1,24 | 1.148 | 0.295 | 0.046 |
| **EXPECTATION** |  | **df** | **F** | **p** | **η²_p_** |
|  | SESSION | 4,24 | 0.137 | 0.895 | 0.006 |
|  | SESSION * STIMULATION | 4,24 | 0.416 | 0.687 | 0.017 |
|  | SESSION * AGE | 4,24 | 0.381 | 0.711 | 0.016 |
|  | SESSION * STIMULATION * AGE | 4,24 | 1.368 | 0.264 | 0.054 |
|  | STIMULATION | 1,24 | 0.718 | 0.405 | 0.029 |
|  | AGE | 1,24 | 0.145 | 0.707 | 0.006 |
|  | STIMULATION * AGE | 1,24 | 1.921 | 0.178 | 0.074 |

# Possible adverse effects of brain stimulation

Possible adverse effects were collected from participants after each stimulation session, together with the likelihood of such effects being caused by stimulation. Table V summarizes the number of participants reporting a side effect, thought to be related to stimulation, with their respective group percentage. Fisher’s exact test did not reveal any significant association between assigned and perceives group allocation (ACTIVE versus SHAM), thus subjects were blind to the stimulation group (p = 0.673).

| Table V Number n (and percentage %) of subjects in the ACTIVE (n=14) and CONTROL (n=14) groups reporting each side effect, in every session   \|  \| **ACTIVE [n (%)] – SHAM [n (%)]** \| \| \| \| \| \| --- \| --- \| --- \| --- \| --- \| --- \| \| **Side effect** \| **SESSION 1** \| **SESSION 2** \| **SESSION 3** \| **SESSION 4** \| **SESSION 5** \| \| HEADACHE \| 1(7)-0(0) \| 0(0)-1(7) \| 1(7)-0(0) \| 0 (0)-1(7) \| 1(7)-0(0) \| \| PAIN IN NECK \| 0(0)-0(0) \| 0(0)-1(7) \| 1 (7)-0(0) \| 0 (0)-0(0) \| 0 (0)-0(0) \| \| SLEEPINESS \| 1(7)-1(7) \| 0(0)-1(7) \| 1(7)-1(7) \| 1(7)-1(7) \| 1(7)-1(7) \| \| ITCHING \| 5(36)-4(29) \| 4(29)-1(7) \| 3(21)-1(7) \| 2(14)-1(7) \| 4(29)-1(7) \| \| TROUBLE CONCENTRATING \| 3(21)-1(7) \| 3(21)-2(14) \| 2(14)-1(7) \| 2(14)-1(7) \| 2(14)-1(7) \| \| ACUTE MOOD CHANGE \| 0(0)-0(0) \| 0(0)-0(0) \| 0(0)-0(0) \| 0(0)-0(0) \| 0(0)-0(0) \| \| FATIGUE \| 2(14)-2(14) \| 1(7)-2(14) \| 0(0)-1(7) \| 3(21)-1(7) \| 1(7)-1(7) \| \| NAUSEA \| 0(0)-0(0) \| 0(0)-0(0) \| 0(0)-0(0) \| 0(0)-0(0) \| 0(0)-0(0) \| \| MUSCLE TWITCH IN FACE OR NECK \| 0(0)-0(0) \| 0(0)-0(0) \| 0(0)-0(0) \| 0(0)-0(0) \| 0(0)-0(0) \| \| TINGLING SENSATION IN HEAD OR SCALP \| 7(50)-11(79) \| 6(43)-8(57) \| 5(36)-5(36) \| 6(43)-7(50) \| 6(43)-7(50) \| \| BURNING SENSATION IN HEAD OR SCALP \| 4(29)-2(14) \| 4(29)-1(7) \| 4(29)-1(7) \| 5(36)-0(0) \| 4(29)-1(7) \| \| EPILEPTIC SEIZURE \| 0(0)-0(0) \| 0(0)-0(0) \| 0(0)-0(0) \| 0(0)-0(0) \| 0(0)-0(0) \| \| UNCOMFORTABLE FEELING (NON-SPECIFIC) \| 0(0)-1(7) \| 0(0)-0(0) \| 0(0)-1(7) \| 0(0)-0(0) \| 0(0)-0(0) \| \| LIGHT FLASHES \| 0(0)-0(0) \| 0(0)-0(0) \| 0(0)-0(0) \| 0(0)-0(0) \| 0(0)-0(0) \| |
| --- | --- | --- | --- | --- | --- | --- | --- | --- | --- | --- | --- | --- | --- | --- | --- | --- | --- | --- | --- | --- | --- | --- | --- | --- | --- | --- | --- | --- | --- | --- | --- | --- | --- | --- | --- | --- | --- | --- | --- | --- | --- | --- | --- | --- | --- | --- | --- | --- | --- | --- | --- | --- | --- | --- | --- | --- | --- | --- | --- | --- | --- | --- | --- | --- | --- | --- | --- | --- | --- | --- | --- | --- | --- | --- | --- | --- | --- | --- | --- | --- | --- | --- | --- | --- | --- | --- | --- | --- | --- | --- | --- | --- | --- | --- | --- | --- |

# Strategy use

***Table VI*** shows the counts of individuals reporting use of a strategy or not, for each age group and stimulation group.

| ***Table VI*** *Count of individuals who developed a strategy in the ACTIVE and SHAM groups for each AGE level. Fisher’s exact p-values of the association between STIMULATION and strategy used are also reported.*   \| **CAPACITY** \| **ACTIVE (YES /NO)** \| **SHAM (YES /NO)** \| **p-value** \| \| --- \| --- \| --- \| --- \| \| OO \| 7/0 \| 1/5 \| 0.005 * \| \| YO \| 6/1 \| 8/0 \| 0.467 \|   * p < .05, ** p < .01, *** p < .001 |
| --- | --- | --- | --- | --- | --- | --- | --- | --- | --- | --- | --- | --- |

1. Shape size was six degrees of visual angle, shapes randomly chosen from a set of ten different shapes from Vanderplas and Garvin (1959). [↑](#footnote-ref-1)
2. In RGB notation: red (255,0 ,0), green (0,255,0), blue (0,0,255), yellow (255,255,0), magenta (255,0,255), cyan (0,255,255), white (255,255,255), black (1,1,1), orange (255,128,0) on a grey background (127,127,127). [↑](#footnote-ref-2)
